# *In Vitro* and *In Vivo* Bactericidal and Antibiofilm Efficacy of Alpha Mangostin Against *Staphylococcus aureus* Persister Cells

**DOI:** 10.3389/fcimb.2022.898794

**Published:** 2022-07-22

**Authors:** LewisOscar Felix, Biswajit Mishra, Rajamohammed Khader, Narchonai Ganesan, Eleftherios Mylonakis

**Affiliations:** Infectious Diseases Division, Alpert Medical School of Brown University, Providence, RI, United States

**Keywords:** alpha mangostin, biofilm, persisters, *Staphylococcus aureus*, *Galleria mellonella*

## Abstract

The formation of persister cells is associated with recalcitrance and infections. In this study, we examined the antimicrobial property of alpha mangostin, a natural xanthone molecule, against methicillin-resistant *Staphylococcus aureus* (MRSA) persisters and biofilm. The MIC of alpha mangostin against MRSA persisters was 2 µg/ml, and activity was mediated by causing membrane permeabilization within 30 min of exposure. The membrane activity of alpha mangostin was further studied by fast-killing kinetics of MRSA persiste r cells and found that the compound exhibited 99.99% bactericidal activity within 30 min. Furthermore, alpha mangostin disrupted established MRSA biofilms and inhibited bacterial attachment as biofilm formation. Alpha mangostin down-regulated genes associated with the formation of persister cells and biofilms, such as *norA*, *norB*, *dnaK*, *groE*, and *mepR*, ranging from 2 to 4-folds. Alpha mangostin at 16 μg/ml was non-toxic (> 95% cell survival) to liver-derived HepG2 and lung-derived A549 cells, similarly. Still, alpha mangostin exhibited 50% cell lysis of human RBC at 16 μg/ml. Interestingly, alpha mangostin was effective *in vivo* at increasing the survival up to 75% (*p*<0.0001) of *Galleria mellonella* larvae infected with MRSA persister for 120 h. In conclusion, we report that alpha mangostin is active against MRSA persisters and biofilms, and these data further our understanding of the antistaphylococcal activity and toxicity of this natural compound.

## Introduction

*Staphylococcus aureus* is a major human pathogen associated with hospital and community-acquired infections and can cause bacteremia and sepsis (Tong et al., 2015; Poolman and Anderson, 2018). The rise in antibiotic resistance has severely repressed the treatment options for *S. aureus* infection (Andersson et al., 2021). Methicillin-resistant *S. aureus* (MRSA) is one of the most common drug-resistant bacteria, responsible for over 60% of *S. aureus* infections in the United States (Okwu et al., 2019). The Centers for Disease Control and Prevention (CDC) has reported more than 90,000 severe cases of MRSA, causing 35,000 deaths each year in the USA (Okwu et al., 2019). Vancomycin is considered the antibiotic of last resort against MRSA. Still, its increased usage has resulted in the emergence of vancomycin-resistant (VRSA) or intermediate *S. aureus* (VISA) strains (Ahmed and Baptiste, 2018).

In addition to the ongoing drug-resistance issue, a significant setback for the failure of antimicrobial therapy is the formation of bacterial persister cells (Chang et al., 2020). Bacteria adapt to a phenotypic dormant transient state with high antibiotic tolerance (Lechner et al., 2012). Persister cells are not genetically inherited but develop when a small group of bacteria subjected to a lethal concentration of antibiotics withstands the antibiotic exposure (Balaban et al., 2004; Brauner et al., 2016; Peyrusson et al., 2020). *S. aureus* tends to develop persisters in the stationary phase of growth (Conlon et al., 2015), and persister *S. aureus* cells tolerate major classes of antibiotics, including ciprofloxacin (DNA replication inhibitor), gentamicin (protein synthesis inhibitor) and vancomycin (cell wall synthesis inhibitor) (Simões et al., 2018).

In a further complex antibiotic tolerant state, *S. aureus* forms biofilms comprised of bacteria inside an extracellular polymeric substance made of polysaccharides, proteins, and e-DNA (Craft et al., 2019). Standard antibiotic treatments are ineffective against biofilm formed by *S. aureus* persisters, leading to complexities, including recurrence of severe infections, such as endocarditis and osteomyelitis (Conlon, 2014). Small molecules targeting the bacterial membrane and cell wall provide a promising strategy to overcome the severe concern of *S. aureus* persister and biofilms (Fisher et al., 2017) and, in previous reports, our group reported that molecules that permeabilize the cell membrane are effective against *S. aureus* persister and biofilms (Kim et al., 2015; Kim et al., 2016; Kim et al., 2018a; Kim et al., 2018b).

In this study, we explored the therapeutic potential of alpha mangostin, a natural xanthone molecule from mangostin fruit (*Garcinia mangostana*) (Koh et al., 2013). While alpha mangostin is known to demonstrate bactericidal activity against Gram-positive bacteria, including *S. aureus* (Koh et al., 2013), the potential to target *S. aureus* persisters and persister regulating gene is unexplored. In this study, we conducted a series of experiments exploring the *in vitro* and *in vivo* activity of alpha mangostin against MRSA persisters.

## Materials and Methods

### Bacterial Strains, Growth Condition, and Persister Cell Isolation

Community-acquired methicillin-resistance *S*. *aureus* (CA-MRSA) strain MW2 BAA-170 was obtained from ATCC (Manassas, VA, USA). To isolate persister cells, overnight cultures of *S. aureus* MW2 (OD600nm) grown in tryptic soy broth (TSB) (BD) at 37°C were treated with 10X MIC (20 μg/ml) of gentamicin for 4 h as described (Allison et al., 2011).

### Antimicrobial Agents and Chemicals

Unless specified, alpha mangostin and other antimicrobial agents were purchased from Sigma Aldrich (MO, USA), and the antibiotics stock solutions were made in DMSO or ddH_2_O, as indicated.

### Minimum Inhibitory Concentration of Alpha Mangostin Against *S. aureus* MW2 Persister Cells

We performed a standard micro-dilution method (CLSI, 2018) to determine the MIC of alpha mangostin against *S. aureus* MW2 persister cells (CLSI, 2018). In short, serial dilution (50 μl) of alpha mangostinwere made in 96-well plates (Cat No. 3595, Corning, NY, USA) containing Mueller-Hinton Broth (MHB) in triplicates. Then, overnight culture of *S. aureus* MW2 was grown to stationary phase and treated with 20 μg/ml of gentamicin for 4 h. We washed the bacterial cells thrice using phosphate-buffered saline (PBS). Then, we added 50 μl of *S. aureus* persister cell (1x10^5^ CFU/ml) to wells containing the dilution of alpha mangostin. The plate was incubated at 37°C for 18 h. After incubation, the plate was read spectrophotometrically at OD_600_ using SpectraMax M3, Molecular Devices (San Jose, CA, USA). The MIC is the drug concentration devoid of any bacterial growth.

### Killing Kinetics of Alpha Mangostin Against *S. aureus* MW2 Persister Cells

We prepared *S. aureus* MW2 persister cells by adding 20 μg/ml of gentamicin to an overnight bacterial culture and incubating for an additional 4 h (Kim et al., 2015). After incubation, we washed the bacterial cells thrice with an equal volume of PBS and set OD_600_ ~ 0.4 (~2 x 10^8^CFU/ml). We added 1 ml of *S. aureus* MW2 persister cells with alpha mangostin (2 μg/ml) and vancomycin (4 μg/ml) inside a 2 ml deep well assay block (Corning Costar 3960). Then, we incubated the plate at 37°C with shaking at 225 rpm. At specific time points (0, 30, 60 and 120 mins), we removed 50 μl of the samples and serially diluted, and spot-plated on tryptic soy agar (TSA, BD) plates enumerating the number of persister cells. All the experiments were done in triplicate.

### Cell Membrane Permeability Assay Against *S. aureus* MW2 Persister Cells

We performed a fluorescence-based cell membrane permeability assay using the protocol established in (Kim et al., 2015). In brief, we cultured overnight cultures of *S. aureus* MW2 to stationary phase and treated them with 20 μg/ml of gentamicin for 4 h. After 4 h, we washed the bacteria thrice with phosphate-buffered saline (PBS) and set OD_600_ ~ 0.4 (~2 x 10^8^ CFU/ml). Then, we added 5 μM of SYTOX Green dye to 10 ml of bacterial cells and incubated the mixture at room temperature for 30 min. In a 96-well plate (Corning catalog no. 3904), we added 50 μl of SYTOX Green/persister cells mixture to50 μl of alpha mangostin (2 μg/ml). Bithionol (2 μg/ml) and vancomycin (4 μg/ml) were used as controls. We measured the fluorescence intensity for 240 min at room temperature using a spectrophotometer (SpectraMax M3, Molecular devices) with excitation and emission wavelengths of 485 and 525 nm, respectively. All the experiments were done in triplicate.

### Checkerboard Assay of Alpha Mangostin With Antibiotics Against *S. aureus* Strain MW2

We performed a checkerboard assay to evaluate the synergy between alpha mangostin and antibiotics (Iluz et al., 2018). In a 96-well microtiter plate, we formed an 8x8 matrix with two-fold serial dilution of alpha mangostin and conventional antibiotics (ciprofloxacin, rifampicin, norfloxacin, gentamicin, and vancomycin). We prepared *S. aureus* MW2 bacteria as described in the MIC experiments and added them to the corresponding wells. Then incubated, the plates at 37°C for 18 h.

After incubation, we read the plates spectrophotometrically at OD_600_ using SpectraMax M3, Molecular Devices (San Jose, CA, USA). We calculated the fractional inhibition concentration index (FICI using the formula FICI=MIC of compound A in combination/MIC of compound A alone + MIC of compound B/MIC of compound B alone. The FICI value is categorized by Synergy – FICI < 0.5, additive – 0.5- 4, and antagonism, FICI > 4 (Iluz et al., 2018).

### Effects of Alpha Mangostin on Initial *S. aureus* MW2 Adhesion

We cultivated an overnight culture of *S. aureus* MW2 in TSB (supplemented with 0.2% glucose and 3% NaCl) media. In a 96-well plate (Cat No. 3903, Corning, NY, USA), we serially diluted 50 μl of alpha mangostin solution (64 -1 μg/ml). To this plate, we added a high density (1.5 OD_600nm_) of overnight culture (*S. aureus* MW2) and incubated at 37°C for 1 h. After incubation, we discarded the media and washed the wells carefully with PBS to remove any loosely attached or free-floating planktonic cells (Mishra et al., 2016; Peng et al., 2021). We added 20% of XTT to the wells after discarding the planktonic cells and incubated the plate for another 2 h at 37^°^C. We quantified the inhibition of initial biofilm adhesion using XTT [2,3- bis(2-methyloxy-4-nitro-5-sulfophenyl)-2H-tertazolium-5-carboxanilide following manufacture instructions with minor adjustments (ATCC, VA, USA). We recorded the absorbance at 450 nm using SpectraMax M2 Multi-mode Microplate Reader (Molecular Devices, CA, USA). We calculate the percentage of biofilm growth assuming 100% biofilm attachment is achieved in the bacterial wells without alpha mangostin treatment.

In addition, to quantitate the effect of alpha mangostin on biomass reduction of initial biofilm adhesion, we performed crystal violet (CV) staining. *S. aureus* initial biofilm adhesion plates were set as described above for the XTT assay. In brief, we incubated the 96-well plates for 1 h at 37°C, discarded the media, and gently washed the wells with phosphate-buffered saline to remove any loosely attached or floating cells. We then added 100 μl of 0.4% CV stain to all the well-containing biofilms and let them stand for 15 min at room temperature. We removed the excess dye by washing twice with sterile water after 15 min. Finally, we added 100 μl of 95% ethanol to dissolve the CV stain and visualized the *S. aureus* biofilm adhesion (LewisOscar et al., 2017).

### Inhibition of *S. aureus* MW2 Biofilm Formation

We tested the efficacy of alpha mangostin in inhibiting biofilm formation by *S. aureus* using the assay described in (Mishra et al., 2016; Peng et al., 2021). We cultivated an overnight culture of *S. aureus* MW2 in TSB (supplemented with 0.2% glucose and 3% NaCl) media. The next day, an exponential phase of bacterial culture was done in the same TSB media and diluted to OD_600_ ~0.03. In a 96-well plate (Cat No. 3903, Corning, NY, USA), we serially diluted 50 μl of alpha mangostin solution (64 -1 μg/ml). To this plate, we added 50 μl of the bacterial culture and incubated the plate at 37°C for 24 h. After incubation, we discarded the media and washed the wells carefully with PBS to remove any loosely attached or free-floating planktonic cells (Mishra et al., 2016; Peng et al., 2021). We added, 20% of XTT to the wells after discarding the planktonic cells and incubated the plate for another 2 h at 37°C. We quantified the inhibition of biofilm formation using XTT [2,3- bis(2-methyloxy-4-nitro-5-sulfophenyl)-2H-tertazolium-5-carboxanilide] following manufacture instructions with minor adjustments (ATCC, VA, USA). We recorded the absorbance at 450 nm using SpectraMax M2 Multi-mode Microplate Reader (Molecular Devices, CA, USA). We calculate the percentage of biofilm growth assuming 100% biofilm attachment is achieved in the bacterial wells without alpha mangostin treatment.

We performed the *S. aureus* biofilm inhibition assay in the presence of alpha mangostin as described in the initial *S. aureus* biofilm adhesion section. We incubated the 96-well plates at 37°C for 24 h, discarded the media, and washed the wells carefully with PBS to remove any loosely attached or free-floating cells. We then added 100 μl of 0.4% CV stain to all the well-containing biofilms and left them for 15 min at room temperature. After 15 min, we removed the excess dye by washing twice with sterile water. We added 95% ethanol to the 96-well plates to release the fixed CV stain and visualized the inhibition of *S. aureus* biofilm formation (LewisOscar et al., 2017).

### Disruption of *S. aureus* MW2 Mature Biofilms

We cultured overnight culture of *S. aureus* MW2 in TSB media (supplemented with 0.2% glucose and 3% NaCl). The next day, we cultured an exponential phase of bacterial cells in the same TSB media and diluted to OD_600_ ~0.03. In a flat bottomed 96-well polystyrene microtiter plate (Cat No. 3903, Corning, NY, USA), we added 100 μl of the adjusted bacterial culture and incubated the plates at 37°C for 24 h in static conditions to allow robust biofilm formation. After incubation, we discarded the media and washed the wells carefully with PBS to remove any loosely attached or free-floating planktonic cells (Mishra et al., 2016; Peng et al., 2021). To the established *S.aureus* MW2 biofilms in 96-well plates, we added serially diluted alpha mangostin (64 -1 μg/ml) in fresh TSB media and incubated the plates for another 24 h. After discarding the media containing planktonic cells and washing the wells with PBS to remove loosely attached or free-floating cells, we added 20% XTT in TSB media to the established *S. aureus* MW2 biofilms in 96-well plates. We incubated the plates for another 2 h at 37°C. We performed colorimetric quantification of the disruption of mature biofilm using XTT [2,3- bis(2-methyloxy-4-nitro-5-sulfophenyl)-2H-tertazolium-5-carboxanilide] following manufacture instructions with minor adjustments (ATCC, VA, USA) and measured the absorbance at 450 nm using SpectraMax M2 Multi-mode Microplate Reader (Molecular Devices, CA, USA). The percentage of biofilm growth was plotted, assuming 100% biofilm growth is achieved in the bacterial wells without alpha mangostin treatment.

We pre-formed *S. aureus* mature biofilm for 24 h and treated the established biofilm with alpha mangostin as mentioned in the initial *S. aureus* biofilm adhesion section. We incubated the 96-well plates for another 24 h at 37°C, discarded the media, and washed the wells carefully with PBS to remove any loosely attached or free-floating cells. We then added 100 μl of 0.4% CV stain to all the well-containing biofilms and left them for 15 min at room temperature. We removed the excess dye by washing twice with sterile water after 15 min. We added 95% ethanol to the 96-well plates to release the fixed CV stain and visualized the disruption of preformed *S. aureus* biofilms (LewisOscar et al., 2017).

### Confocal Laser Scanning Microscopy of Alpha Mangostin Treated *S. aureus* MW2 Biofilm (Formation and Disruption) Using Live/Dead Staining

We performed a confocal microscopic analysis of alpha mangostin treated and untreated *S. aureus* biofilms at two different stages (biofilm formation and established biofilm). To visualize the effect of alpha mangostin on inhibition of biofilm formation, we inoculated 1 ml of exponential phase culture of *S. aureus* MW2 (10^6^ CFU/ml) in TSB media (supplemented with 0.2% glucose and 3% NaCl) along with alpha mangostin (2 μg/ml final concentration) inside a chamber cuvette (Borosilicate cover glass system, Nunc Cat No:155380). After incubation for 24 h at 37°C, we discarded the media and washed the chambers carefully with PBS to remove any loosely attached or free-floating cells (Mishra et al., 2016). We processed the control biofilm similarly except for the addition of alpha mangostin.

According to the manufacturer’s instructions, we stained *S. aureus* MW2 biofilms using a LIVE/DEAD staining kit (Invitrogen Molecular Probes, USA). The biofilm was observed using a 63X oil immersion objective (63X/1.2 W) in a confocal laser scanning microscope (CLSM) (Model: Zeiss 880) (Carl Zeiss, Germany). The excitation/emission was set at 555 nm/>550 nm for propidium iodide and 488 nm/<550 nm for SYTO9. The 2D, ortho, and 3D images for 24 and 48 h of alpha mangostin treated and untreated *S. aureus* MW2 biofilms were acquired using Zen black software (Carl Zeiss, Germany). The acquired Z-stack images were processed using Zen Blue lite software (Carl Zeiss, Germany) (Mishra et al., 2016).

Similarly, to visualize the effect of alpha mangostin on disruption of established biofilms, we inoculated 1 ml of exponential phase culture of *S. aureus* MW2 (10^6^ CFU/ml) in TSB media (supplemented with 0.2% glucose and 3% NaCl) inside a chamber cuvette (Borosilicate cover glass system, Nunc Cat No:155380). We incubated the chambers for 24 h at 37°C to allow biofilm formation. After incubation, we discarded the media and washed the chambers carefully with PBS to remove any loosely attached or free-floating cells. We added 1 ml fresh TSB to this pre-formed biofilm with 4 μg/ml of alpha mangostin and incubated the cuvette for another 24 h. We processed the control biofilm similarly except for the addition of alpha mangostin. After the incubation, we stained the biofilms like the biofilm formation assay and visualized them under the confocal microscope (Mishra et al., 2016).

### Reverse Transcriptase Real-Time PCR

We performed RT-PCR-based transcription to quantitate the impact of alpha mangostin on the MRSA persister genes (Khader et al., 2020). In brief, we prepared *S. aureus* MW2 persister cells by adding 20 μg/ml of gentamicin to an overnight bacterial culture and incubating for an additional 4 h. After incubation, we washed the bacterial cells thrice with an equal volume of PBS and diluted the bacterial cells to OD600 ~ 0.4. Cells were treated with 1/2X MIC (Sub-MIC) of alpha mangostin and equimolar vancomycin concentration for 1 h. After incubation, we centrifuged the bacterial cell and washed the cells with PBS. Then, we performed RNA isolation and purification using RNeasy mini kit (Qiagen, Hilden, Germany) following the manufacturer’s instructions. Using the Verso cDNA synthesis kit, we used the purified RNA for cDNA synthesis using the Verso cDNA synthesis kit (Thermo Fisher Scientific, MA, USA). We used the primers reported by (Dawan et al., 2020) **(** **Table S1** **)** for RT-PCR. Further, we performed the RT-PCR (iCycler iQ real-time detection system, Bio-Rad) by adding 5 μl of cDNA, 12.5 μl SYBR Green Master Mix (Bio-Rad), 2 μl of primers (5 mM), and 3.5 μl RNase free water (Ambion, St. Austin, TX, United States). The conditions for RT-PCR cycles were 95◦C for 30 s; 40 cycles at 95°C for 5 s; 55°C for 30 s; finishing with a melt curve analysis from 65 to 95°C (Khader et al., 2020). We calculated the relative expression ratio using the calculation as follows: n-fold expression = 2^-ΔΔCt^, ΔΔCt = ΔCt (drug-treated)/ΔCt (untreated), where ΔCt represents the difference between the cycle threshold (Ct) of the gene studied and the Ct of housekeeping 16S rRNA gene (internal control) (Mitchell et al., 2012).

### Mammalian Cell Cytotoxicity Assays

Liver-derived HepG2 and lung-derived A549 cells were used to assess the mammalian cell cytotoxicity of alpha mangostin using previously described methods (Kim et al., 2018b; Peng et al., 2021). The cells were grown in Dulbecco’s Modified Eagle Medium (DMEM) (Gibco, MD, USA) supplemented with 10% fetal bovine serum (FBS) (Gibco, MD, USA) and 1% penicillin/streptomycin (Gibco, MD, USA) and maintained at 37°C in 5% CO_2_. Cells were harvested and resuspended in a fresh medium, and 100 μl were distributed in a 96-well plate at 1 × 106 cells/well. Alpha mangostin were serially diluted in serum, antibiotic-free DMEM added to the monolayer of the cells, and the plates were incubated at 37°C in 5% CO_2_ for 24 h. At 4 h, before the end of the incubation period, 10 μl of 2-(4-iodophenyl)-3- (4-nitrophenyl)-5-(2, 4-disulfophenyl)-2H-tetrazolium (WST-1) solution (Roche, Mannheim, Germany) was added to each well. WST-1 reduction was monitored at 450 nm using a using SpectraMax M2 Multi-mode Microplate Reader (Molecular Devices, CA, USA). Assays were performed in triplicate, and the percentage of cell survival was calculated. The lethal dose (LD_50_) is considered the concentration of compounds responsible for killing 50% of cells (Musa et al., 2010).

### Hemolysis of Human Red Blood Cells

The hemolytic activity of alpha mangostin was tested towards haemoglobin based on a modified method (Lin et al., 2020). Human erythrocytes were purchased from Rockland Immuno- chemicals (Limerick, PA, USA), washed thrice with equal volume PBS and made 4% hRBCs. In 96-well microtiter well plate, 100 μl of RBCs were added to 100 μl of alpha mangostin serially diluted in PBS from 128-4 μg/ml. 1% of Triton-X 100 was used as the positive control, and PBS alone was used as a negative control. The plate was incubated for 1 h at 37°C. After incubation, centrifuged at 500x g for 5 min. One hundred microlitres of supernatant were transferred to a fresh 96 well microtiter well plate, and the OD was measured at 540 nm. The percentage of hemolysis was calculated using 100% hemolysis caused by 1% Triton X-100. The values were represented as the mean of duplicates. The following formula calculated the percentage of hemolysis: (A540 nm in the peptide solution − A540 nm in PBS)/(A540 nm of 1% Triton X-100 treated sample − A540 nm in PBS) × 100 (Peng et al., 2021). Zero and 100% percentage hemolysis were determined in PBS and 0.1% Triton X-100, respectively.

### Calculation of Therapeutic Index (MHC/MIC Ratio):

The value of the Therapeutic index (TI) represents the property of cell selectivity of a drug molecule. TI is a measurement of the capability of distinctions between any pathogen and its host cells. The TI is calculated as the ratio of MHC (minimum hemolytic activity) and MIC (minimum inhibitory concentration) (Chen et al., 2005). We did not observe any detectable hemolysis at 8 μg/mL of alpha mangostin. So, we used the MHC value of 16 μg/ml to calculate the TI.

### Galleria Mellonella Assays

To study the protective effects of the alpha mangostin in an *in vivo* model, we used an established wax moth model system (Peleg et al., 2009; Desbois and Coote, 2011). *G. mellonella* larvae (Vanderhorst Wholesale, St. Mary’s, OH, USA) were distributed in a different group with randomly selected larvae (n = 16/group). *S. aureus* MW2 persister cells were prepared by adding 20 μg/ml of gentamicin to an overnight bacterial culture and incubated for an additional 4 h. After incubation, the bacterial cells were washed thrice with an equal volume of PBS and were diluted to OD_600_ ~ 0.3. Experimental groups consisted of untouched (no injection), PBS (vehicle), bacterial infection, and treatment groups. Each group was injected with 10 μl (2 × 106 cells/ml) of the prepared bacteria, followed by alpha mangostin treatment (32-4 mg/kg doses) after 1 h. Vancomycin (25 mg/kg) was used as a positive control. All injections were performed using a Hamilton syringe (Merck, Darmstadt, Germany) on the last left proleg. The larvae were incubated at 37°C for 5 days, with live and dead counts performed every 24 h.

### Statistical Analysis

We used the Student t-test for statistical analysis of biofilms and cell cytotoxicity. One-way ANOVA for qPCR analysis. *p* < 0.05 was considered a significant difference. For the *G. mellonella* survival experiment, we plotted the Kaplan–Meier curves using GraphPad Prism Version 6.04 (GraphPad Software, La Jolla, CA). We used the same program to perform statistical analysis and plotting of *p < 0.05* value.

## Results

### Bactericidal Activity of Alpha Mangostin Against *S. aureus* MW2 Persisters

**Figure 1:**
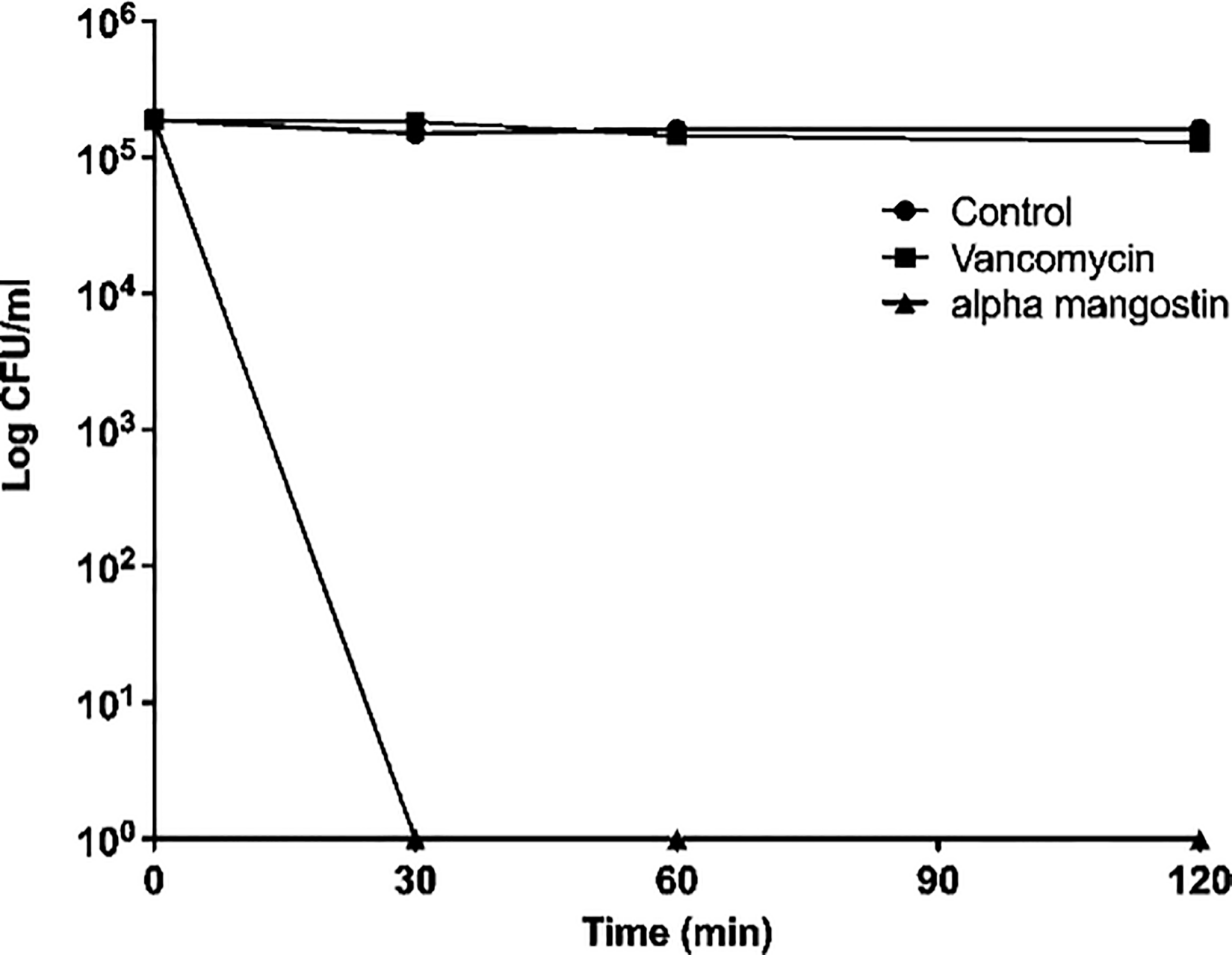
Killing kinetics. Growth curves were generated using *S. aureus* MW2 persister cells. Colony-forming units were measured by plating the treated and untreated bacterial cells on a TSA plate after serial dilution. Data represent the mean ± SD (n = 3).

We first tested the antibacterial activity of alpha mangostin against *S. aureus* MW2 persisters and found that the MIC of alpha mangostin against *S. aureus* MW2 persisters was 2 μg/ml. Also, we performed time-kill kinetics of alpha mangostin against MRSA persisters at 1X MIC. Alpha mangostin exhibited rapid bactericidal activity and, within 30 mins of exposure. We assigned the 99.99% elimination of MRSA to denote no colonies of *S. aureus* MW2 persister on TSB plates upon exposure of 2 µg/ml of alpha mangostin for 30 mins (**Figure 1**). In contrast, vancomycin (at 4 μg/ml) was ineffective in decreasing the bacteria counts up to 120 mins.

### Alpha Mangostin Selectively Disrupts *S. aureus* MW2 Persister Cell Membrane

**Figure 2:**
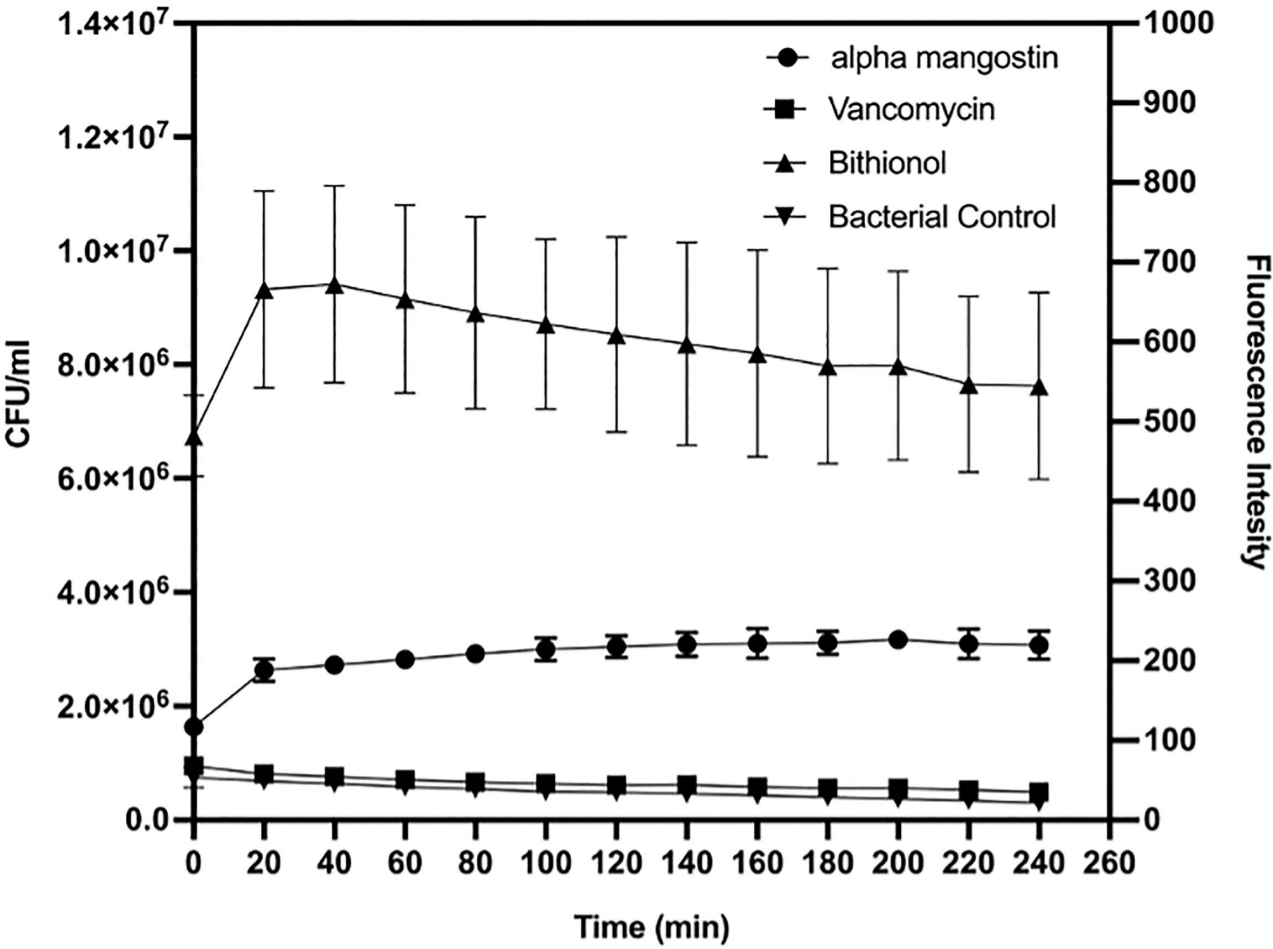
Time course membrane permeabilization by alpha mangostin (2 μg/ml), vancomycin (4μg/ml), and bithionol (2 μg/ml) against *S. aureus* MW2 persisters. Membrane permeabilization was measured spectrophotometrically by monitoring the SYTOX green dye uptake (excitation wavelength of 485 nm and an emission wavelength of 530 nm). Data represent the mean ± SD (n = 3).

As shown in **Figure 2**, alpha mangostin induced rapid membrane permeabilization at the MIC concentration (2 μg/ml). The relative fluorescence for persister cells was 220 units and 540 units for alpha mangostin and bithionol, respectively. Notably, the relative fluorescence for normal cells (non-persister cell) was 600 units and 900 units for alpha mangostin and bithionol, respectively **(** **Figure S1** **)**.

### Alpha Mangostin Enhances Antibiotics Activity

We performed a checkerboard titration assay to evaluate the interaction of alpha mangostin with conventional antibiotics (**Table S2**). The FICI index of mupirocin (0.5), rifampicin (1.03), ciprofloxacin (2.1), gentamicin (2.5), and vancomycin (3.0) suggests exerting an additive effect. In contrast, norfloxacin was an antagonist with a FICI index of > 5. The results of the checkerboard assay gave an insight into using alpha mangostin in combination with antibiotics to improve the efficacy against MRSA persister and biofilm.

### Alpha Mangostin Inhibits Different Stages of *S. aureus* MW2 Biofilm

**Figure 3:**
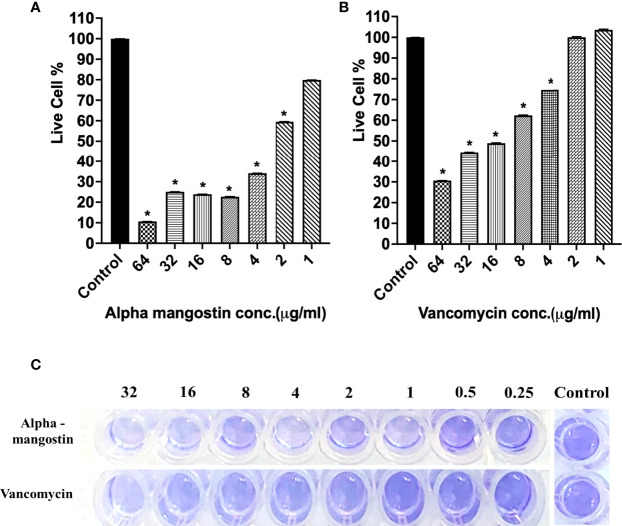
**(A, B)** *S. aureus* MW2 initial biofilm adhesion in 96-well microtiter plate for 1 h treated with alpha mangostin and vancomycin at concentrations ranging from 64-1 µg/ml. Live cells % in the treated and non-treated initial biofilm adhesion quantified using XTT at OD_450_. Results are shown as means ± s.d.; n=3 (*p < 0.05). **(C)** Visual depiction of the initial biofilm adhesion with CV staining in 96-well microtiter plate after treatment with alpha mangostin and vancomycin at comparable concentrations.

To check the antibiofilm efficacy of alpha mangostin, we conducted antibiofilm assays evaluating the inhibition of the three different stages of *S. aureus* MW2 biofilms (initial attachment, formation, and disruption of established biofilm) (**Figure 3**). We tested the effects of alpha mangostin from concentrations ranging from 64-1 μg/ml against all three biofilm stages (initial adhesion, biofilm formation, and established biofilms of *S. aureus* MW2). We included vancomycin as antibiotic control for comparison at a similar concentration range. At 64 μg/ml concentration, alpha mangostin reduced the initial bacteria attachment by 90% (**Figures 3A, B** **)**. Live cell quantification of biofilm adhesion using XTT showed that alpha mangostin at 4 μg/ml inhibited more than 75% of the *S. aureus* MW2 initial bacterial adhesion. Further, CV staining provided visual confirmation of the efficacy of alpha mangostin in preventing initial adhesion of *S. aureus* MW2 biofilms in 96-well plates **(** **Figure 3C** **)**.

**Figure 4:**
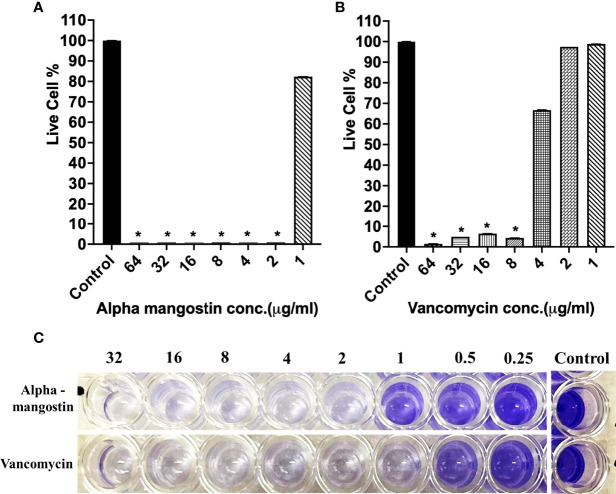
**(A, B)** *S. aureus* MW2 biofilm formation in 96-well microtiter plate for 24 h treated with alpha mangostin and vancomycin at concentrations ranging from 64-1 µg/ml. Live cells % in the treated and non-treated biofilm formation were quantified using XTT at OD_450_. Results are shown as means ± s.d.; n=3 (*p < 0.05). **(C)** Visual depiction of the biofilm formation with CV staining in 96-well microtiter plate after treatment with alpha mangostin and vancomycin at comparable concentrations.

In the case of inhibiting *S. aureus* MW2 biofilm formation, alpha mangostin at 2 μg/ml reduced the percentage of live cells to less than 15%, while vancomycin was effective at 1 μg/ml concentration (**Figures 4A, B** **)**. The lowest concentration of any test compound required to inhibit maximum (>50%) biofilm formation is considered as minimum biofilm inhibitory concentration (MBIC) (Cruz et al., 2018). Using live-cell quantification by XTT, we found that alpha mangostin at 2 μg/ml inhibited more than >50% of the *S. aureus* MW2 biofilms at its formation stage **(** **Figure 4A** **)**. In agreement, CV staining also showed a gradual increase of violet color depicting more biomass in wells treated with lower concentrations of alpha mangostin (up to 1 μg/ml) **(** **Figure 4C** **)**. Therefore, we determined the MBIC value for alpha mangostin as 2 μg/ml against *S. aureus* MW2 biofilm formation.

**Figure 5:**
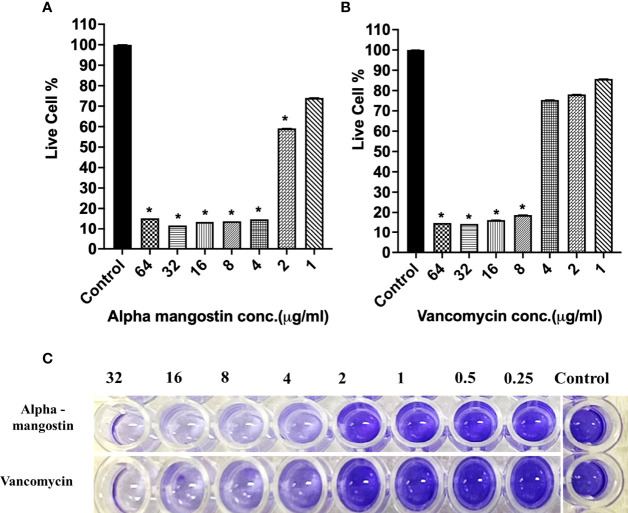
**(A, B)** *S. aureus* MW2 mature biofilm in a 96-well microtiter plate for 24 h treated with alpha mangostin and vancomycin at concentrations ranging from 64-1 µg/ml. Live cells % in the treated and non-treated *S. aureus* MW2 mature were quantified using XTT at OD_450_. Results are shown as means ± s.d.; n=3 (*p < 0.05). **(C)** Visual depiction of the *S. aureus* MW2 mature with CV staining in 96-well microtiter plate after treatment with alpha mangostin and vancomycin at comparable concentrations.

Using XTT quantification, we determined the minimum biofilm eradicating concentration (MBEC) of alpha mangostin against 24 h established biofilms of* S. aureus MW2*. The MBEC value is determined as the lowest concentration of any test compound required to eradicate maximum (>50%) established biofilms (Cruz et al., 2018). Alpha mangostin at twice its MIC (4 μg/ml) eradicated 96% of established *S. aureus *biofilms, while at 4 μg/ml, it only reduced 40% of the biofilms (**Figures 5A**). In comparison, at a similar concentration of 4 μg/ml, vancomycin only reduced 18% of the established biofilms (**Figures 5B** **)**. Thus, we determined the MBEC value of alpha mangostin as 4 μg/ml against *S. aureus* MW2 biofilm formation. Further, we used CV staining to confirm the disruption of *S. aureus* mature biofilm at 4 μg/ml of alpha mangostin **(** **Figure 5C** **)**.

### Confocal Microscopic Analysis of Alpha Mangostin Inhibiting and Disrupting MRSA Biofilm

**Figure 6:**
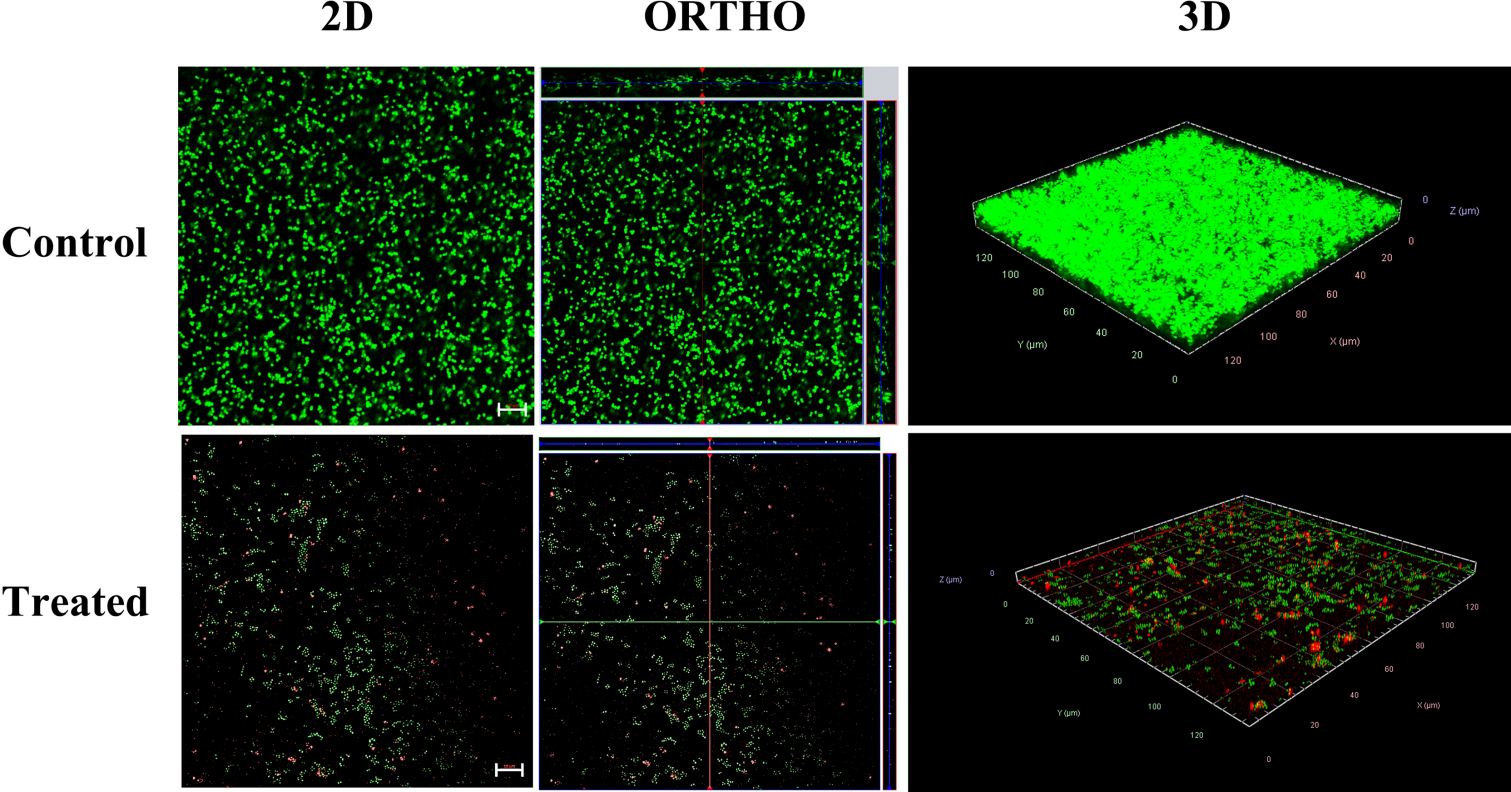
Confocal microscopic images of the *S. aureus* MW2 biofilm formation treated with alpha mangostin (2 μg/ml). *S. aureus* MW2 biofilm treated and untreated with alpha mangostin was stained with SYTO-9 dye that stains live cells green and propidium iodide (PI) that stains dead cells red. Scale bars correspond to 10 µm.

**Figure 7:**
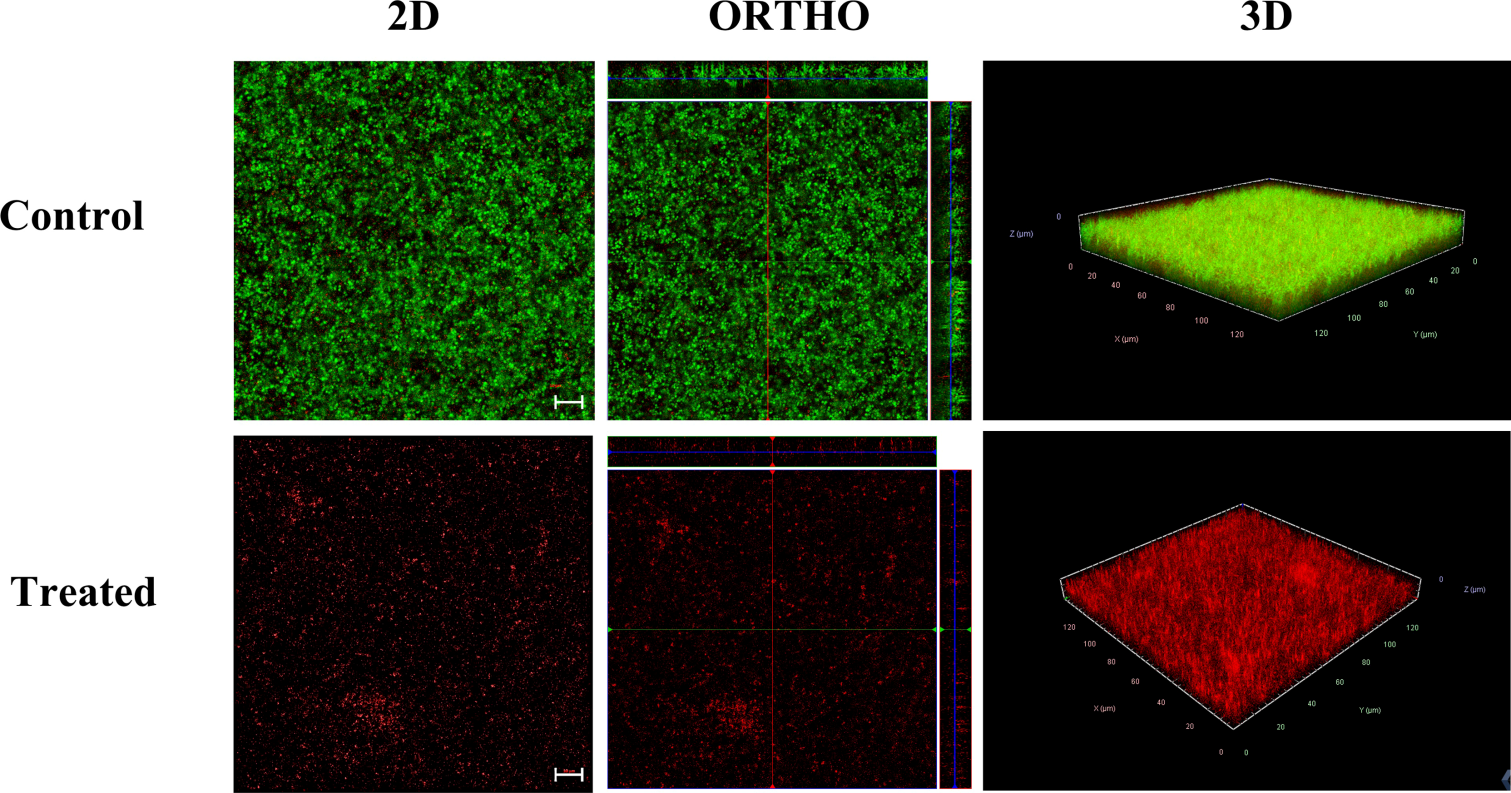
Confocal microscopic images of the *S. aureus* MW2 mature biofilm treated with alpha mangostin (2 μg/ml). *S. aureus* MW2 mature biofilm treated and untreated with alpha mangostin was stained with SYTO-9 dye that stains live cells green and propidium iodide (PI) that stains dead cells red. Scale bars correspond to 10 µm.

To visualize the effect of alpha mangostin on biofilm formation and mature biofilm stages of *S. aureus* MW2, we performed confocal microscopic analysis. We treated the biofilms with 2 μg/ml of alpha mangostin corresponding to its MBIC value to inhibit biofilm formation. Similarly, for established *S. aureus* MW2 biofilms, we used 4 μg/ml of alpha mangostin corresponding to its MBEC concentration. In a LIVE/DEAD staining, most cells, including live cells, uptakes SYTO9 and fluoresce green, while PI penetrates the dead cells and fluoresce red. Interestingly, the 2D, Ortho, and 3D images of alpha mangostin for treated MRSA biofilms at both stages showed; reduced bacterial biomass in the biofilm matrix, less biofilm thickness, and the presence of red color indicative of dead cells in the *S. aureus* MW2 biofilm (**Figures 6**, **7**).

### Alpha Mangostin Down Regulating Genes Associated With the Formation of the *S. aureus* Persisters and Biofilms Gene

**Figure 8:**
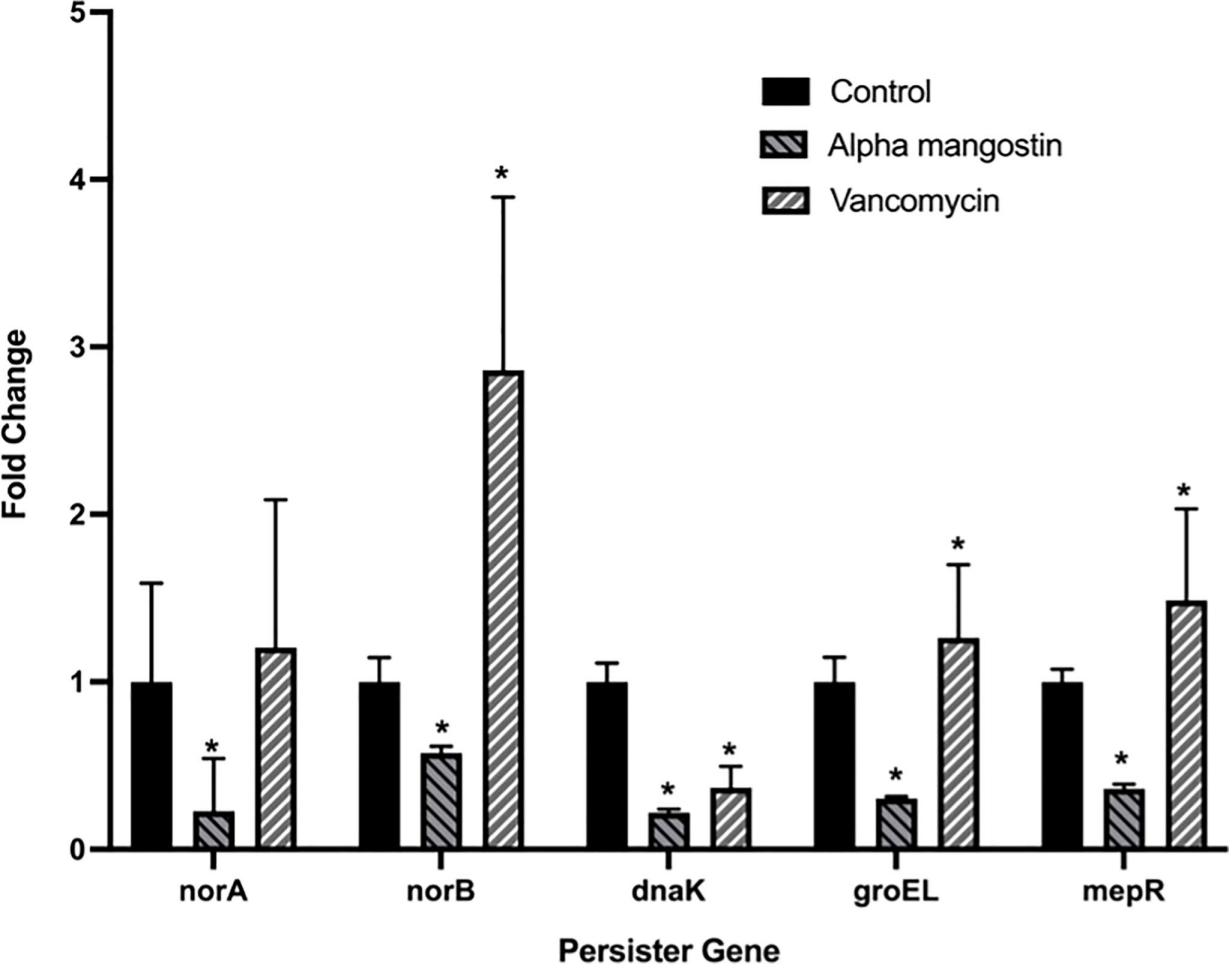
Alpha mangostin down-regulates genes associated with the formation of MRSA persister and biofilms. *S. aureus* MW2 persister treated with a sub-MIC concentration of alpha mangostin for 4h. Exposure of alpha mangostin was associated with the down-regulation of persister and biofilm-forming genes. Data represent the mean ± SD (n = 3). *p < 0.05 analyzed by One-way ANOVA.

We tested the efficacy effect of alpha mangostin to down-regulate the gene involved in *S. aureus* persister formation (**Figure 8**). Four important genes, including *norA*, *norB*, *dnaK*, *groEL* and *mepR* were tested at the transcriptional level. We treated the MRSA persister with sub- MIC concentration (1 μg/ml) of alpha mangostin. As control, we used vancomycin at equimolar concentrations. Interestingly, alpha mangostin down-regulated all tested genes and significantly down-regulated *norA* and *dnaK* by 4-fold, *groEL*, and *mepR* by 3-fold, and norB by 2-fold (*p*<0.0001). On the other hand, vancomycin upregulated *norB*, *groEL*, and *mepR* and down-regulated *dnaK*.

### Alpha Mangostin Toxicity to Mammalian Cell Lines, Red Blood Cells and *G. mellonella* Wax Moth

**Figure 9:**
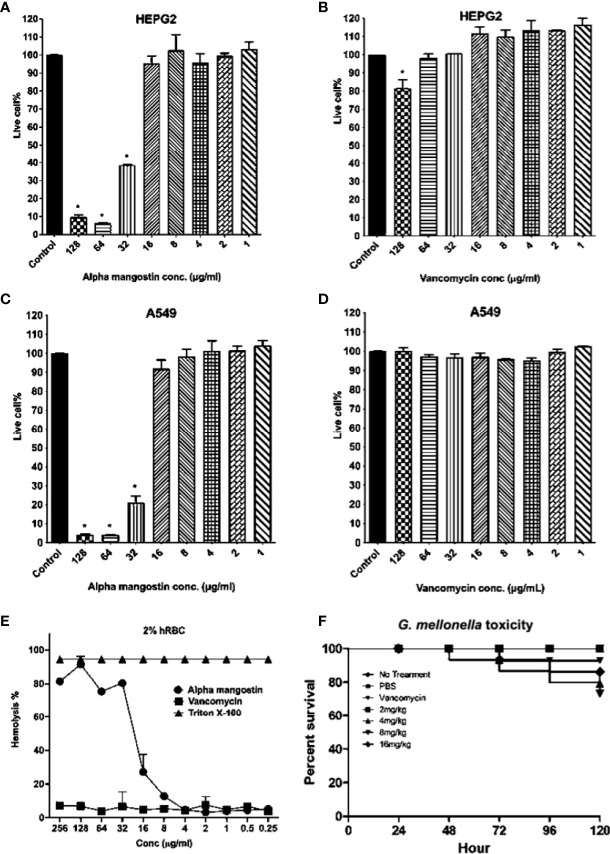
Cytotoxicity, hemolytic, and *in vivo G. mellonella* toxicity of alpha mangostin. **(A–D)** Cellular toxicity of alpha mangostin against liver-derived HEPG2 and lung-derived A549 cell lines showed that the LD_50_ values (p < 0.05) as16 μg/ml. **(E)** Hemolysis of human blood red cells (hRBCs) showed the HL_50_ of alpha mangostin was 16 μg/ml. Vancomycin was used as a negative control and Triton-X (0.0025-1%) was used as a positive control **(F)** The toxicity of alpha mangostin tested in *G. mellonella* model showed that >70% of larvae survived at the higher concentration of 16 mg/kg until 120 h (*p*<0.05). Data represent the mean ± SD (n = 3) for 5 A-E and n=16 larvae per group for 5F.

We tested the toxicity of alpha mangostin to human cell lines, red blood cells and a whole-body *Galleria mellonella* wax moth model (**Figure 9**). The LD_50_ value of alpha mangostin and vancomycin against HEPG2 cells was found to be < 32 μg/ml (only 40% of cells survived at this concentration) and >128 μg/ml, respectively (**Figures 9A, B** **)**. For A549 cell lines, only 22% of cells survived at 32 μg/ml of alpha mangostin, while 100% of cells survived up after exposure to vancomycin at 128 μg/ml of vancomycin (**Figures 9C, D** **)**. We further evaluated the toxicity of alpha mangostin to human red blood cells (**Figure 9E**). The alpha mangostin was found to cause 50% human red blood lysis (HL_50_) at a concentration of 16 μg/ml, which is higher than 5 times the MIC values against *S. aureus* persister. We also tested the toxicity of alpha mangostin in a live wax moth larvae model (**Figure 9F**). We found that 80% of *G. mellonella* larvae survived alpha mangostin up to 120 h (*p=*0.001). But at the higher concentration of 16 mg/Kg of alpha mangostin, only 20% of *G. mellonella* larvae died.

### Therapeutic Index of Alpha Mangostin

The therapeutic index is commonly used to identify the specificity of antimicrobial agents. A higher therapeutic index value indicates greater antimicrobial specificity. In our study, the MHC of alpha mangostin against mammalian erythrocytes was 16 μg/ml, while the MIC against *S. aureus *MW2 was 2 μg/ml. Thus, calculating the ratio of MHC and MIC gives the TI value of alpha mangostin as 8 **(** **Table 1** **)**.

**Table 1:** Minimum inhibitory concentration, Minimal hemolytic concentration and therapeutic index of alpha mangostin against *S.aureus* MW2.

Bacterial Strain	MIC	MHC	Therapeutic Index
*S. aureus* MRSA MW2	2	16	4

### Alpha Mangostin Enhances Antibiotics Activity

Because of toxicity, alpha mangostin may need to be evaluated in combination with other antibiotics and we performed a checkerboard titration assay to evaluate the interaction of alpha mangostin with conventional antibiotics (**Table S2**). The FICI index of mupirocin (0.5), rifampicin (1.03), ciprofloxacin (2.1), gentamicin (2.5) and vancomycin (3.0) suggests exerting an additive effect.

### Alpha Mangostin Rescues MRSA Persister Infected *G. mellonella* Wax Moth

**Figure 10:**
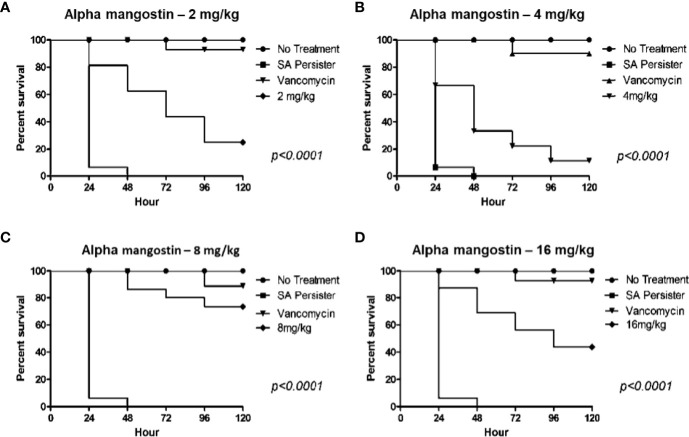
*In vivo* activity of alpha mangostin against *S. aureus* MW2 persisters in the *G. mellonella* wax moth model. **(A–D)**. Sixteen *S. aureus* persister infected *G. mellonella* were treated with control (PBS), 25 mg/kg vancomycin and 2, 4, 8, 16 mg/kg alpha mangostin at 1 h of post infection. Seventy five percentage of larvae survived with treatment of 8 mg/kg of alpha mangostin was significant compared to PBS treatment (*p*<0.0001).

We also evaluated the *in vivo* efficacy of alpha mangostin in preventing the killing of *G. mellonella* by MRSA persisters (**Figure 10**). In order to confirm that injected MRSA cells were in a persister state, we isolated the MRSA from the larva after incubation for 24 h and tested the MIC of the isolated MRSA against vancomycin. The MIC of vancomycin was > 64 μg/ml, validating the persister state. Interestingly, alpha mangostin improved the survival of *G. mellonella* larvae infected with MRSA persister cells up to 16 mg/kg. A dose of 8 mg/kg rescued 75% of larvae (*p*<0.0001). The lower concentrations of alpha mangostin (2 and 4 mg/kg) were less effective, while the reduced survival of *G. mellonella* larva at 16 mg/kg was attributed to probable toxicity induced by alpha mangostin.

## Discussion

*S. aureus* causes acute and recalcitrant infections due to the emergence of antibiotic tolerant and metabolically dormant persister cells (Chambers and DeLeo, 2009; Tong et al., 2015; Poolman and Anderson, 2018; Kim et al., 2019). The treatment of *S. aureus* infections is often complicated due to the ability of the bacteria to form persister cells (Conlon, 2014). Key strategies to eradicate *S. aureus* persister cells include metabolic stimuli that enable the killing of bacteria by antibacterial agents such as aminoglycosides (Allison et al., 2011), and the use of compounds that target the bacterial membrane (Kim et al., 2019). In this context, our findings demonstrated that alpha mangostin penetrates MRSA persister cell membranes (**Figure 2**) and interferes with the development of MRSA biofilms. Mangosteen (*Garcinia mangostana*) is a common tropical fruit in Southeast Asia, India, and Sri Lanka that is used in traditional medicine for the treatment of skin infections, chronic wounds, and dysentery (Pedraza-Chaverri et al., 2008). Xanthones derivatives are the major bioactive molecules of mangostin that display potential antibacterial, antifungal, anti-inflammatory, anticancer, antioxidant, and anti-allergy properties (Pedraza-Chaverri et al., 2008; Obolskiy et al., 2009). A specific xanthone derivative that exhibits antibacterial properties is alpha mangostin (1,3,6-trihydroxy-7-methoxy-2,8-bis(3-methylbut-2-enyl)xanthen-9-one) derived from mangostin pericarp extracts (Nguyen and Marquis, 2011). Koh et al. (2013) used fluorescent dyes to demonstrate that alpha mangostin causes membrane disruptions leading to leakage of intracellular contents in *S. aureus* and MRSA. Interestingly, this work demonstrated that the hydrophobic association of alpha mangostin with the lipid membrane of* S. aureus *causes membrane deformation and diffusion of water molecules (Koh et al., 2013). Later, Phuong et al. (2017) provided additional evidence on the alteration of the staphylococcal membrane by alpha mangostin. However, these reports did not study the effects of this compound against staphylococcal persister cells, and our findings expand these previous reports by demonstrating the rapid bactericidal activity of alpha mangostin against *S. aureus* persister cells **(** **Figures 1**, **2** **)** and we found that alpha mangostin effectively down-regulates the gene involved in *S. aureus* persister formation (**Figure 8** **)**. Furthermore, previous studies reported that alpha mangostin works only against the preformed biofilms of *S. aureus * (Phuong et al., 2017; Nguyen et al., 2021). Then, we use the *G. mellonella* model to demonstrate that alpha mangostin is effective against *S. aureus* persister cells during infection **(** **Figure 10** **)**. To our knowledge, this is the first report on the effects of alpha mangostin on *S. aureus* persister cells under *in vitro* and *in vivo* conditions. In this report, we report that alpha mangostin inhibits and disrupts the formation of *S. aureus* biofilms at all three stages (initial adhesion, formation, and maturation) **(** **Figures 3**–**5** **)**. According to Phuong et al. (2017), alpha mangostin inhibited MRSA biofilm formation but did not affect the mature biofilms of MRSA. Our study expands this finding by demonstrating the effects of alpha mangostin in various stages of *S. aureus* biofilm formation **(** **Figures 3** **–** **5** **)**. Confocal microscopic evidence revealed that alpha mangostin reduces the thickness of *S. aureus* biofilm at various stages (biofilm formation and mature biofilm).

MRSA persister and biofilm are tolerant to antimicrobial agents and show reduced metabolic activity (Waters et al., 2016). In *S. aureus*, the formation of both persister cells and biofilm is mediated by genes such as *norA*, *norB*, *dnaK*, *groEL, *and* mepR* (Arita-Morioka et al., 2015; abd El-Baky, 2016; Dawan et al., 2020). More specifically, *norA* and *norB* are associated with antimicrobial resistance and tolerance in biofilm (Soto, 2013) and *norA* is associated with resistance against hydrophilic fluoroquinolones (ciprofloxacin and norfloxacin), while *norB* regulates efflux pumps induced by both hydrophobic and hydrophilic fluoroquinolones (Costa et al., 2013). Additionally, both *dnaK* and *groEL *encode molecular chaperones that regulate persister cell and biofilm formation (Arita-Morioka et al., 2015; abd El-Baky, 2016; Dawan et al., 2020), while *mepR* belongs to the multidrug and toxic compound transporter family and is associated with repression of the multidrug transporter (MepA) (Schindler and Kaatz, 2016; Dawan et al., 2020).

Toxicity analysis is an important step in drug discovery for determining the drug concentration, controlled exposure, and duration of exposure to both animals and humans. We tested the toxicity of alpha mangostin in the mammalian cell membrane, sheep red blood cells (RBCs), and a *G. mellonella* model. Alpha mangostin exhibited dose-dependent toxicity to the mammalian cell membrane and sheep RBCs, suggesting that the therapeutic window is relatively narrow. Nevertheless, alpha mangostin was active in the *G. mellonella* model which is widely used to evaluate drug toxicity and efficacy of antibacterial agents (Cutuli et al., 2019). We opted to use the wax moth model since it is well established in determining the survival rate of larva after infection with *S. aureus*  (Khader et al., 2020; Kim et al., 2020). The *G. mellonella* model is widely used to determine the pathogenicity and virulence of multiple pathogens, including MRSA and *S. aureus*  (Peleg et al., 2009; Desbois and Coote, 2011). We also tested alpha mangostin in *G. mellonella* larva after infecting them with MRSA persister cells.

In conclusion, we report that alpha mangostin is effective against MRSA persister cells and biofilm. The high antimicrobial efficacy, fast killing kinetics, membrane targeting, anti-persister, and anti-biofilm characteristics of alpha mangostin suggest that the compound merits further study for the management of topical infections caused by MRSA. Importantly, the toxicity profile of alpha mangostin must be further assessed before this agent can be evaluated for systemic administration.

## Data Availability Statement

The original contributions presented in the study are included in the article/**Supplementary Material**. Further inquiries can be directed to the corresponding author.

## Author Contributions

LF, BM, RK and NG designed and carried out experiments. EM directed research, provided insightful discussions and contributed to the writing. All authors analyzed results, revised the manuscript, and approved of the final version

## Funding

This study was supported by National Institutes of Health Grant P01 AI083214 to EM. This work was supported by P20GM121344 from the National Institute of General Medical Sciences, which funds the Center for Antimicrobial Resistance and Therapeutic Discovery to BM.

## Conflict of Interest

The authors declare that the research was conducted in the absence of any commercial or financial relationships that could be construed as a potential conflict of interest.

## Publisher’s Note

All claims expressed in this article are solely those of the authors and do not necessarily represent those of their affiliated organizations, or those of the publisher, the editors and the reviewers. Any product that may be evaluated in this article, or claim that may be made by its manufacturer, is not guaranteed or endorsed by the publisher.
