# Supplementary figures and images for: In Vitro and In Vivo Bactericidal and Antibiofilm Efficacy of Alpha Mangostin Against Staphylococcus aureus Persister Cells
Source: Front Cell Infect Microbiol. 2022 Jul 22;12:898794. doi: 10.3389/fcimb.2022.898794 (PMC9353584; doi:10.3389/fcimb.2022.898794)

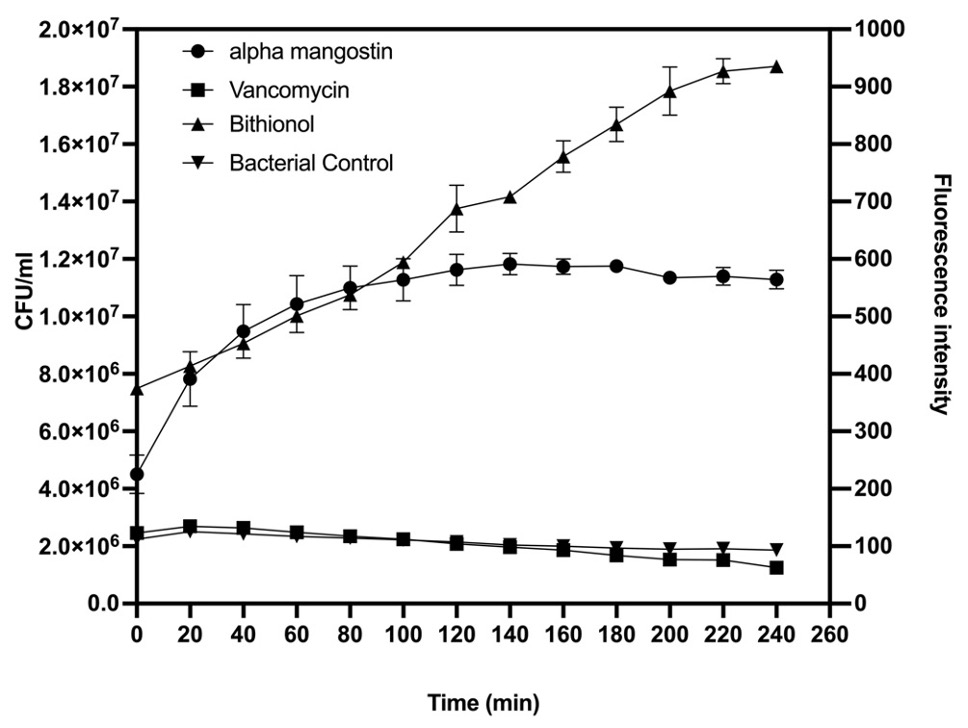

Supplement: Supplementary file 1 [file Image_1.jpeg]
