# Supplementary material for: In Vitro and In Vivo Bactericidal and Antibiofilm Efficacy of Alpha Mangostin Against Staphylococcus aureus Persister Cells
Source: Front Cell Infect Microbiol. 2022 Jul 22;12:898794. doi: 10.3389/fcimb.2022.898794 (PMC9353584; doi:10.3389/fcimb.2022.898794)
Supplement: Supplementary file 2 [file Table_1.docx]

**Table S1.** qPCR primers of genes associated with bacterial *S. aureus* persister and biofilm genes

| **Gene** | **Role of the gene** | **Primer sequence** |
| --- | --- | --- |
| **16S rRNA** | Reference gene | F: CATGCTGATCTACGATTACT  R: CCATAAAGTTGTTCTCAGTT |
| ***dnaK*** | Chaperone proteins | F: ACTTCGTCCGGGTTTACTCC  R: ACAATGGAACCTACACGCCA |
| ***groEL*** | Chaperone proteins | F: CAGTACCACCACCTGCAACA  R: TGCAGCAAGTGAAACAGAGC |
| ***mepR*** | Efflux pump regulator | F: TCGATGCACAAGATACGAGA  R: GCGATACGAGTGTTTGTTCC |
| ***norA*** | Multidrug efflux pump | F: TCGTCTTAGCGTTCGGTTTA  R: TCCAGTAACCATCGGCAATA |
| ***norB*** | Multidrug efflux pump | F: AGCGCGTTGTCTATCTTTCC  R: GCAGGTGGTCTTGCTGATAA |

**Table S2.** Checkerboard assay to analysis the FICI value of alpha mangostin with antibacterial agents

| **SL.No** | **Antibacterial agents** | **FICI** |
| --- | --- | --- |
| 1 | Ciprofloxacin | 2.125 |
| 2 | Rifampicin | 1.031 |
| 3 | Norfloxacin | 5.03 |
| 4 | Gentamicin | 2.5 |
| 5 | Vancomycin | 3 |
| 6 | Mupirocin | 0.527 |
